# Supplementary material for: Microbial diversity drives carbon use efficiency in a model soil
Source: Nat Commun. 2020 Jul 23;11:3684. doi: 10.1038/s41467-020-17502-z (PMC7378083; doi:10.1038/s41467-020-17502-z)
Supplement: Supplementary file 1 — Supplementary Information [file 41467_2020_17502_MOESM1_ESM.pdf]

Microbial diversity drives carbon use efficiency in a model soil.

Domeignoz-Horta et al.

Supplementary Methods 1

Protocol for 16S rRNA amplicon library preparation and sequencing

Briefly, PCR amplicon libraries targeting the 16S rRNA encoding gene present in metagenomic DNA are produced using a barcoded primer set adapted for the Illumina MiSeq(1). DNA sequence data is then generated using Illumina paired-end sequencing at the Environmental Sample Preparation and Sequencing Facility (ESPSF) at Argonne National Laboratory. Specifically, the V4 region of the 16S rRNA gene is PCR amplified with region-specific primers (515F: GTGCCAGCMGCCGCGGTAA; 806R: GGACTACHVGGGTWTCTAAT) that include sequencer adapter sequences used in the Illumina flowcell (1, 2). The forward amplification primer also contains a twelve base barcode sequence that supports pooling of up to 2,167 different samples in each lane(1,2). Each 25 µL PCR reaction contains 9.5 µL of MO BIO PCR Water (Certified DNA-Free), 12.5 µL of QuantaBio's AccuStart II PCR ToughMix (2x concentration, 1x final), 1 µL Golay barcode tagged Forward Primer (5 µM concentration, 200 pM final), 1 µL Reverse Primer (5 µM concentration, 200 pM final), and 1 µL of template DNA. The conditions for PCR are as follows: 94 °C for 3 minutes to denature the DNA, with 35 cycles at 94 °C for 45 s, 50 °C for 60 s, and 72 °C for 90 s; with a final extension of 10 min at 72 °C to ensure complete amplification. Amplicons are then quantified using PicoGreen (Invitrogen) and a plate reader (Infinite® 200 PRO, Tecan). Once quantified, volumes of each of the products are pooled into a single tube so that each amplicon is represented in equimolar amounts. This pool is then cleaned up using AMPure XP Beads (Beckman Coulter), and then quantified using a fluorometer (Qubit, Invitrogen). After quantification, the molarity of the pool is determined and diluted down to 2 nM, denatured, and then diluted to a final concentration of 6.75 pM with a 10% PhiX spike for sequencing on the Illumina MiSeq. Amplicons are sequenced on a 151bp x 12bp x 151bp MiSeq run using customized sequencing primers and procedures(1).

Protocol for ITS amplicon library preparation and sequencing

Genomic DNA was amplified using a custom barcoded ITS primer set, adapted for the Illumina MiSeq (ITS1F: AATGATACGGCGACCACCGAGATCTACAC; ITS2: CAAGCAGAAGACGGCATACGAGAT)(3). The reverse amplification primer also contained a twelve base barcode sequence that supports pooling of up to 2,167 different samples in each lane(1). Each 25ul PCR reaction contains 12ul of MoBio PCR Water (Certified DNA-Free), 10ul of 5 Prime HotMasterMix (1x), 1ul of Forward Primer (5uM concentration, 200pM final), 1ul Golay Barcode Tagged Reverse Primer (5uM concentration, 200pM final), and 1ul of template DNA. The conditions for PCR are: 94°C for 3 minutes to denature the DNA, with 35 cycles at 94 °C for 45 s, 50 °C for 60 s, and 72 °C for 90 s; with a final extension of 10 min at 72 °C to ensure complete amplification.

Amplicons were quantified using PicoGreen (Invitrogen) and a plate reader. Once quantified, different volumes of each of the products are pooled into a single tube so that each amplicon is represented equally. This pool is then cleaned up using the UltraClean® PCR Clean-Up Kit (MoBIO), and then quantified using the Qubit (Invitrogen). After quantification, the molarity of the pool is determined and diluted down to 2nM, denatured, and then diluted to a final concentration of 6.75pM with a 10% PhiX spike for sequencing on the Illumina MiSeq.

References:

1. Caporaso JG*, et al.* (2012) Ultra-high-throughput microbial community analysis on the Illumina HiSeq and MiSeq platforms. *The ISME Journal* 6(**8**):1621-1624.
2. Caporaso JG*, et al.* (2011) Global patterns of 16S rRNA diversity at a depth of millions of sequences per sample. *Proceedings National Academy of Sciences* **1**:4516-4522.
3. Smith DP, Peay KG (2014) Sequence Depth, Not PCR Replication, Improves Ecological Inference from Next Generation DNA Sequencing. *PLoS ONE* 9(**2**): e90234.

Supplementary Table 1. Structural equation model standardized coefficients and specific *P*-value for each path coefficient corresponding to a response and predictor variables. Bacterial community structure: axis 1 of NMDS; Bacterial diversity (PD): bacterial phylogenetic diversity index; Fungi presence: presence/absence of fungi; Fungal:Bacterial ratio: 16S rRNA gene copy number g_{-1} soil: ITS gene copy number g_{-1} soil; EEA/Biomass: maximum activity recorded for Betaglucosidase/ Microbial biomass carbon; CUE: carbon use efficiency.

Response	Predictor	Standardized Coefficient	P value
Bacterial diversity (PD)	Temperature	-0.0735	0.358
Bacterial diversity (PD)	Moisture	-0.0193	0.808
Bacterial diversity (PD)	Fungi presence	0.1744	0.029
Bacterial community structure	Temperature	0.257	0.0005
Bacterial community structure	Moisture	0.3926	< 0.0001
Bacterial community structure	Fungi presence	0.0819	0.259
Fungi presence	Temperature	-0.146	0.177
Fungi presence	Moisture	-0.032	0.761
Fungal:Bacterial ratio	Bacterial community structure	0.109	0.172
Fungal:Bacterial ratio	Bacterial community structure	-0.2742	0.002
Fungal:Bacterial ratio	Fungi presence	0.0582	0.467
Fungal:Bacterial ratio	Temperature	0.0436	0.596
Fungal:Bacterial ratio	Moisture	0.1719	0.046
EEA/Biomass	Bacterial diversity (PD)	-0.0373	0.646
EEA/Biomass	Bacterial community structure	-0.1752	0.032
EEA/Biomass	Fungal:Bacterial ratio	-0.0278	0.733
EEA/Biomass	Fungi presence	0.076	0.349
Aggregation score	Bacterial diversity (PD)	-0.1291	0.059
Aggregation score	Bacterial community structure	-0.2684	0.0006
Aggregation score	Fungi presence	0.1712	0.012
Aggregation score	Fungal:Bacterial ratio	-0.0686	0.319
Aggregation score	Temperature	-0.1875	0.008
Aggregation score	Moisture	0.5223	< 0.0001
CUE	Bacterial diversity (PD)	0.3307	< 0.0001
CUE	Bacterial community structure	-0.379	< 0.0001
CUE	Fungal:Bacterial ratio	-0.0264	0.713
CUE	EEA/Biomass	-0.2119	0.003
CUE	Moisture	0.0354	0.689
CUE	Temperature	0.1264	0.089
CUE	Aggregation score	0.1742	0.041
CUE	Fungi presence	0.1624	0.025

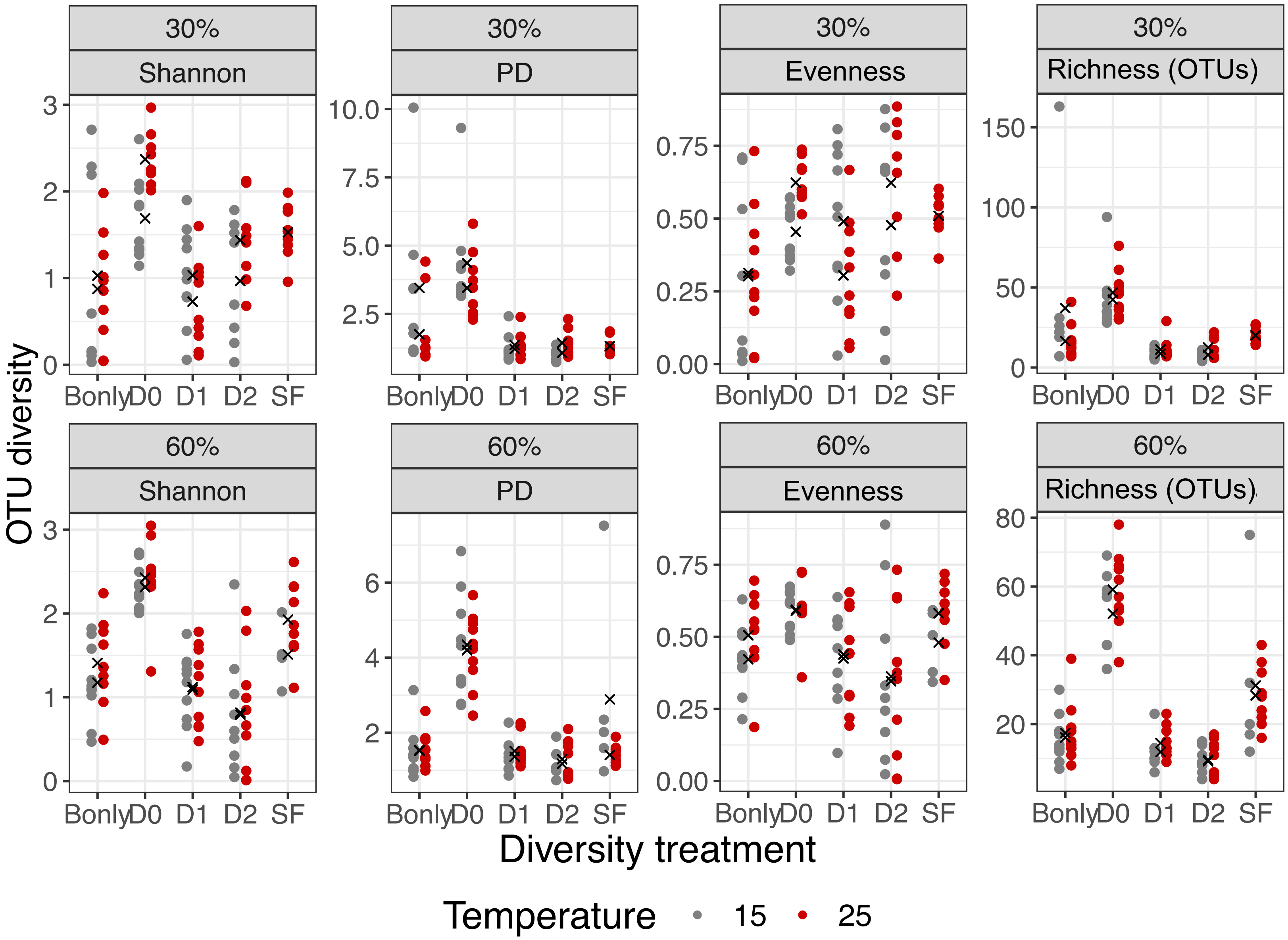

Supplementary Figure 1. Bacterial alpha diversity for each diversity and abiotic treatment. Shannon H, evenness (Pielou.J), phylogenetic diversity (PD) and richness (observed OTUs) diversity metrics are represented to 30% and 60% water holding capacity for the different diversity treatments. Soils incubated at 15ºC and 25ºC are represented in gray and red, respectively. The median for each treatment is indicated by a black cross.

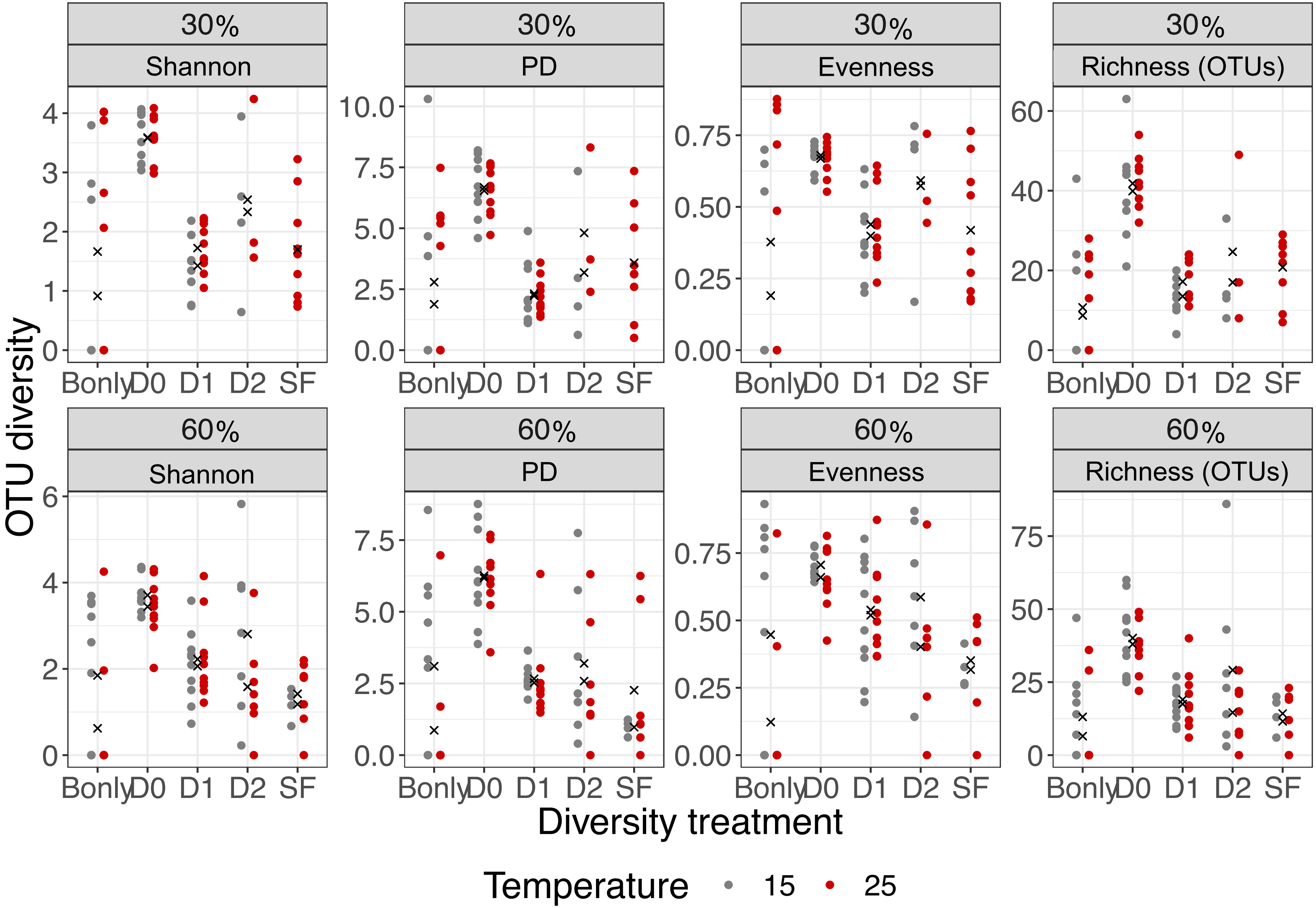

Supplementary Figure 2. Fungal alpha diversity for each diversity and abiotic treatment. Shannon, evenness (Pielou.J), phylogenetic diversity (PD) and richness (observed OTUs) diversity metrics are represented to 30% and 60% water holding capacity for the different treatments. Soils incubated at 15℃ and 25 ℃ are represented in gray and red, respectively. The median for each treatment is indicated by a black cross.

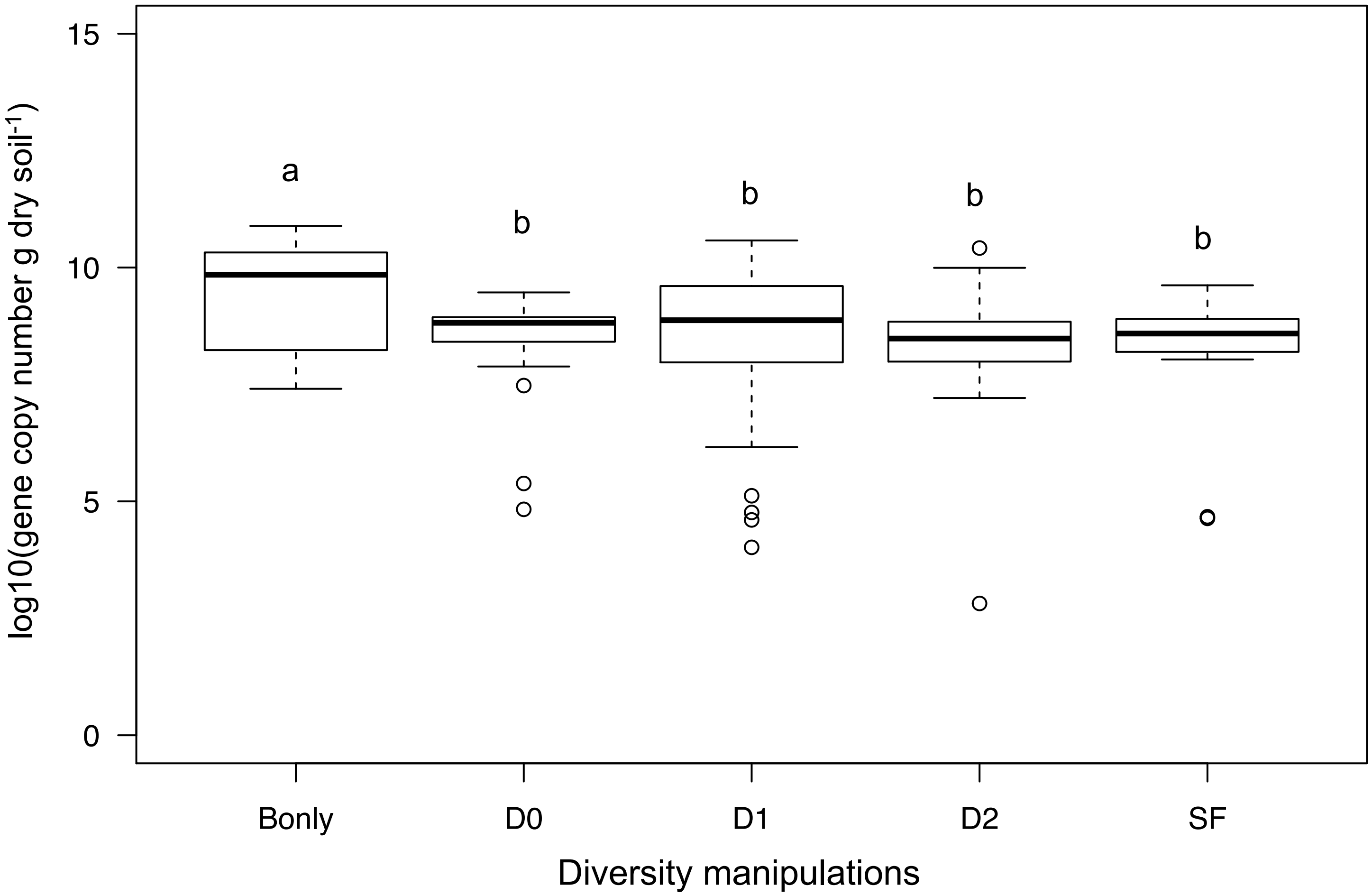

Supplementary Figure 3. Bacterial abundance in the model soil. 16S rRNA gene copy number by treatment. Significant differences between treatments are indicated by different letters (one-way ANOVA followed by Tukey HSD test, $P < 0.05$, df =170, n = 175). In the boxplots, whiskers denote the minimum value or 1.5x interquartile range (whichever is more extreme), and box denotes interquartile range. The horizontal line denotes the median. The number of samples was 40 for D0, D1 and D2, 38 for B_{only} and 20 for SF.

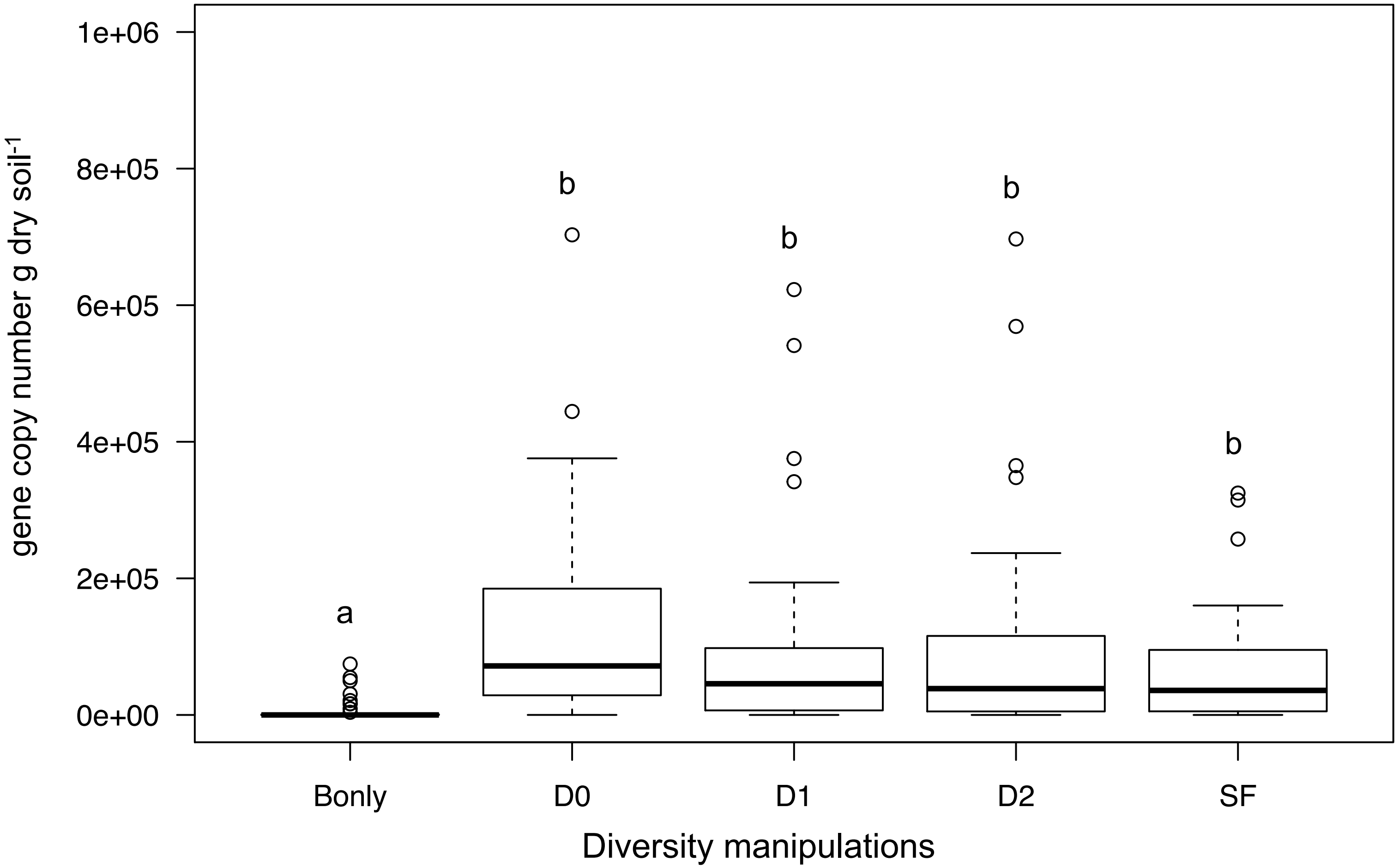

Supplementary Figure 4. Fungal abundance in the model soils. ITS gene copy number by treatment. Significant differences between treatments are indicated by different letters (one-way ANOVA followed by Tukey HSD test, $P < 0.05$, df = 170, n = 175). In the boxplots, whiskers denote the minimum value or 1.5x interquartile range (whichever is more extreme), and box denotes interquartile range. The horizontal line denotes the median. The number of samples was 40 for D0 and D1, 34 for D2, 38 for B_{only} and 23 for SF.

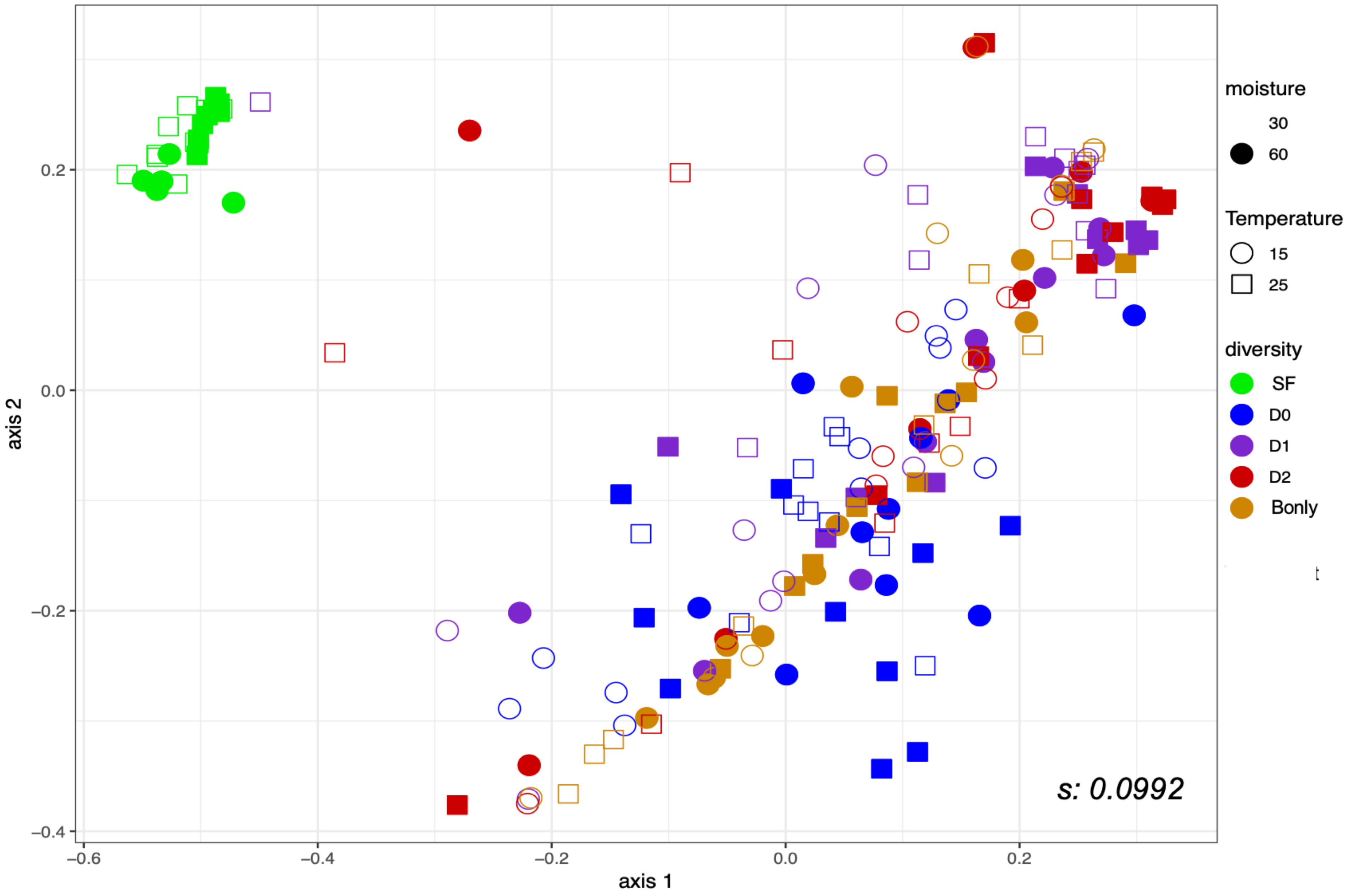

Supplementary Figure 5. Bacterial community structure in the model soils. NMDS ordination of 16S rRNA gene weighted unifrac distance matrices. Samples incubated at different moisture are represented by a empty (30%) or full (60%) symbol, while different temperatures are represented by a circle (15℃) or a square (25℃) and the different diversity treatments are shown in different colors: SF (green), D0 (blue), D1 (violet), D2 (red) and B_{only} (brown). Stress value is indicated at the bottom right.

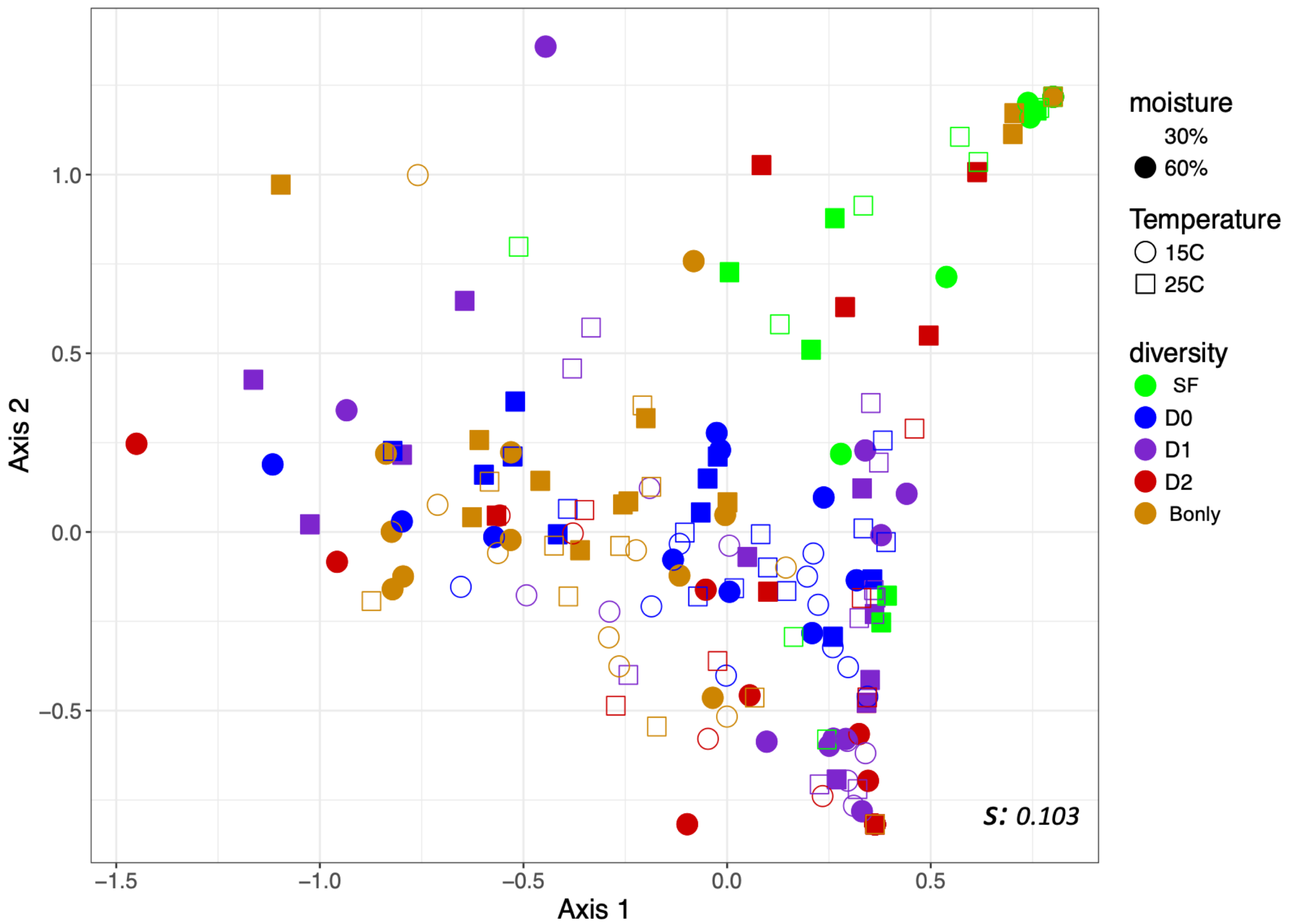

Supplementary Figure 6. Fungal community structure in the model soils. NMDS ordinations of Hellinger distance. Samples incubated at different moisture are represented by an empty (30%) or full (60%) symbol, while different temperatures are represented by a circle (15℃) or a square (25℃) and the different diversity treatments are shown in different colors: SF (green), D0 (blue), D1 (violet), D2 (red) and B_{only} (brown). Stress value is indicated at the bottom right.

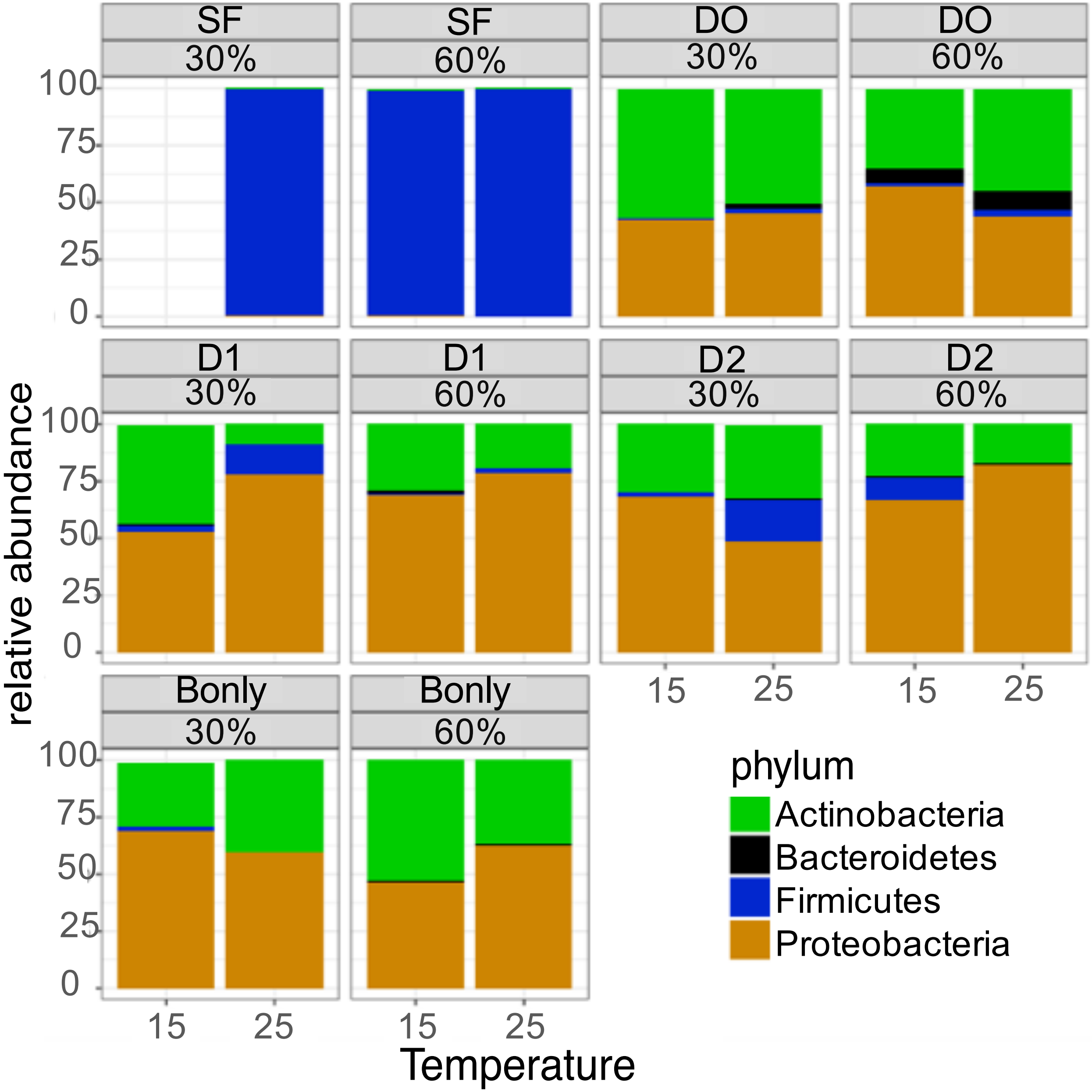

Supplementary Figure 7. Relative abundance of bacterial sequences within phylum, for diversity manipulation, moisture and temperature treatments. Treatment average values for different phylum are shown with different colors: Actinobacteria (green), Bacteroidetes (black) Firmicutes (blue) and Proteobacteria (brown). The number of samples was 20 for D0-30% and D0-60%, 20 for D1-30% and D1-60%, 16 for D2-30%, 19 for D2-60%, 18 for B_{only} -30%, 20 for B_{only} -60% and 9 for SF-30% and 14 for SF-60%.

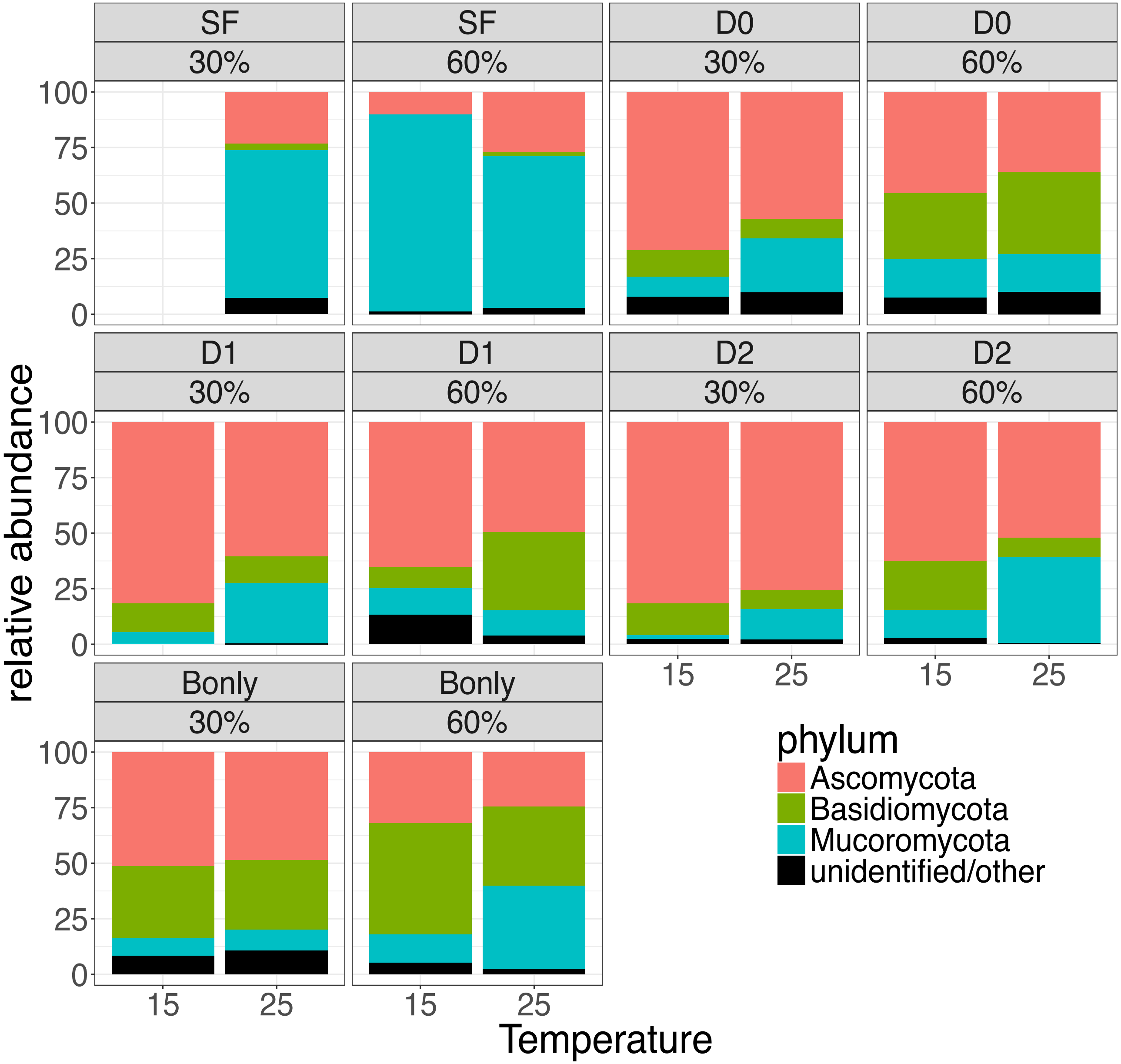

Supplementary Figure 8. Relative abundance of fungal sequences within phylum, for diversity manipulation, moisture and temperature treatments. Treatment average values for different phylum are shown with different colors: Ascomycota (pink), Basidiomycota (green), Mucoromycota (blue) and unidentified (black). The number of samples was 20 for D0-30% and D0-60%; 20 for D1-30% and D1-60%; 4 for D2-30%, 20 for D2-60%, 20 for B_{only} -30% and B_{only} -60% and 9 for SF-30% and 11 for SF-60%.

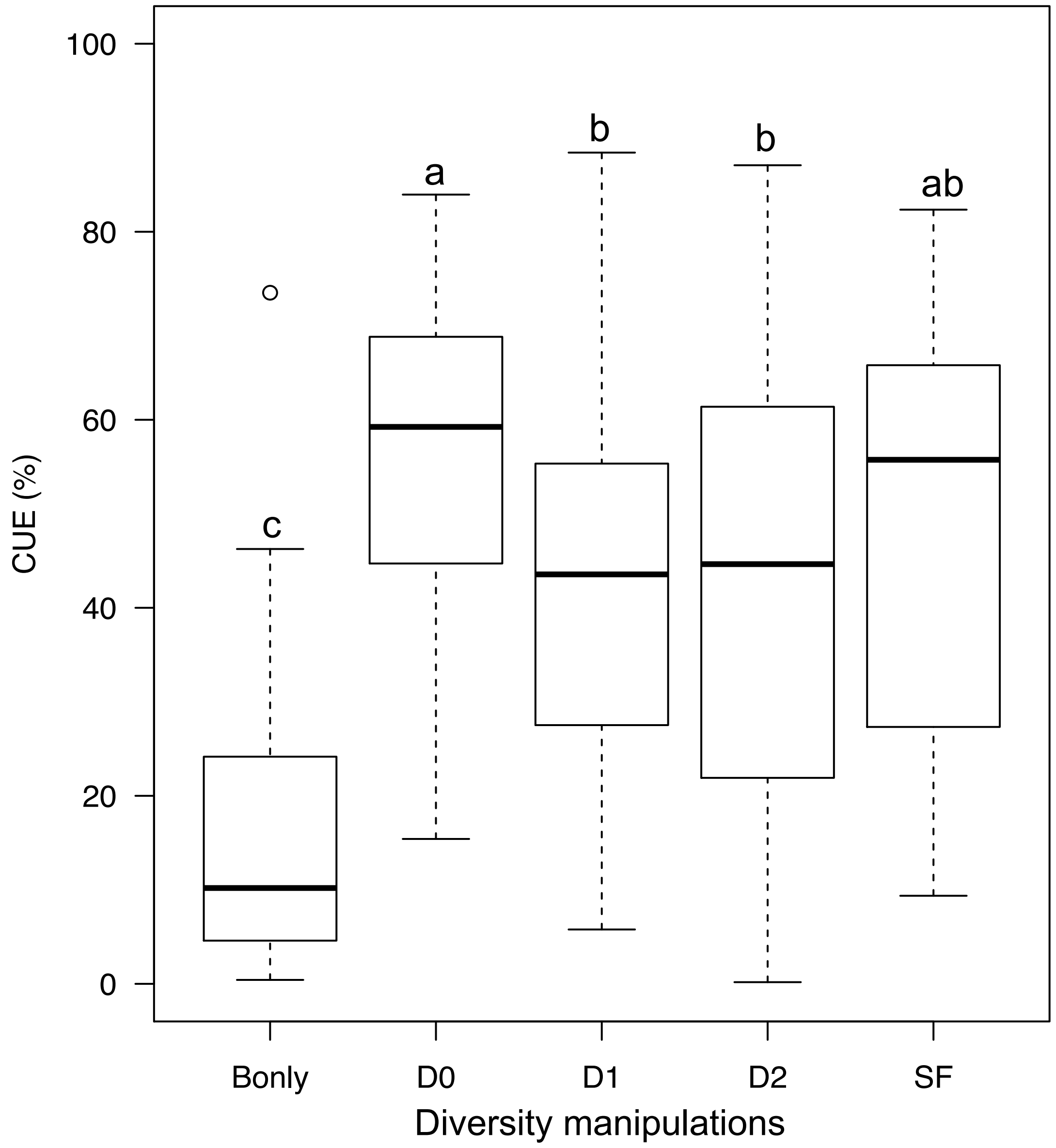

Supplementary Figure 9. Carbon Use Efficiency for each diversity treatment. Significant differences between treatments are indicated by different letters (one-way ANOVA followed by Tukey HSD test, $P < 0.05$, df = 157, n = 162). In the boxplots, whiskers denote the minimum value or 1.5x interquartile range (whichever is more extreme), and box denotes interquartile range. The horizontal line denotes the median. There were 40 samples for D0, 39 for D1, 31 for D2, 32 for B_{only} and 20 for SF.

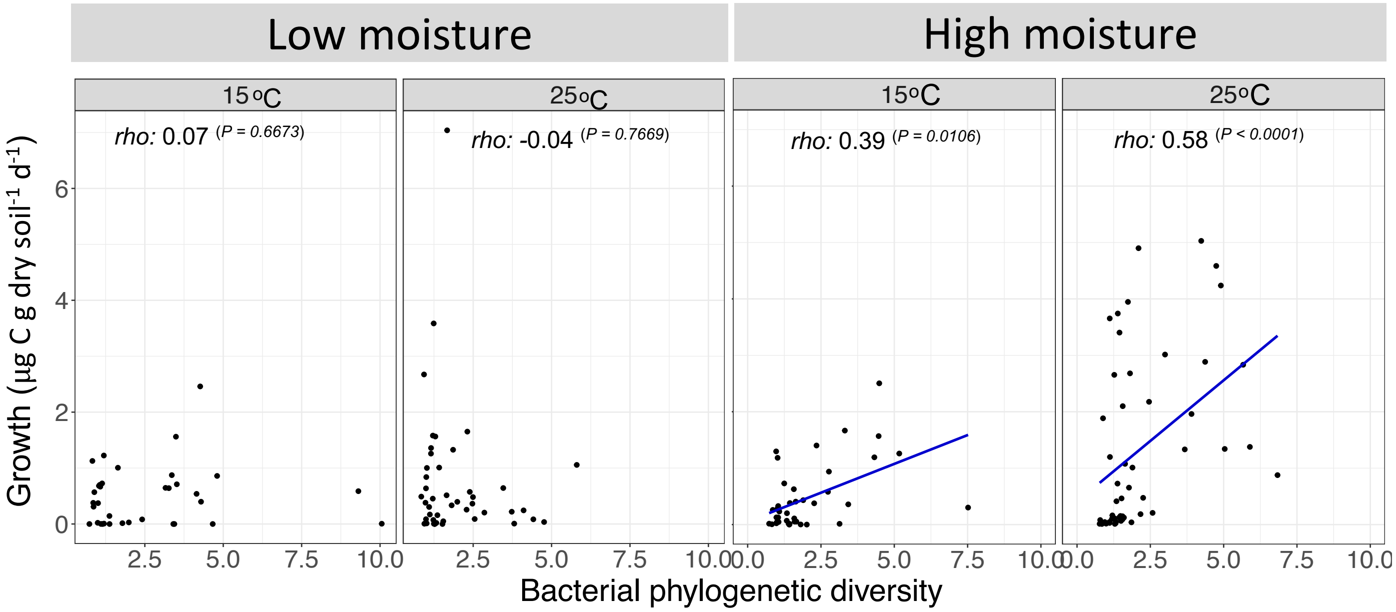

Supplementary Figure 10. Relationship between growth and bacterial phylogenetic diversity at different temperatures for low (30% WHC) and high moisture (60% WHC). Significant relationship are evaluated with spearman correlation and shown in blue when significant. The number of samples was 40 for 15ºC - 30%; 49 for 25ºC - 30%; 43 for 15ºC - 60% and 50 for 25ºC - 60%.

.

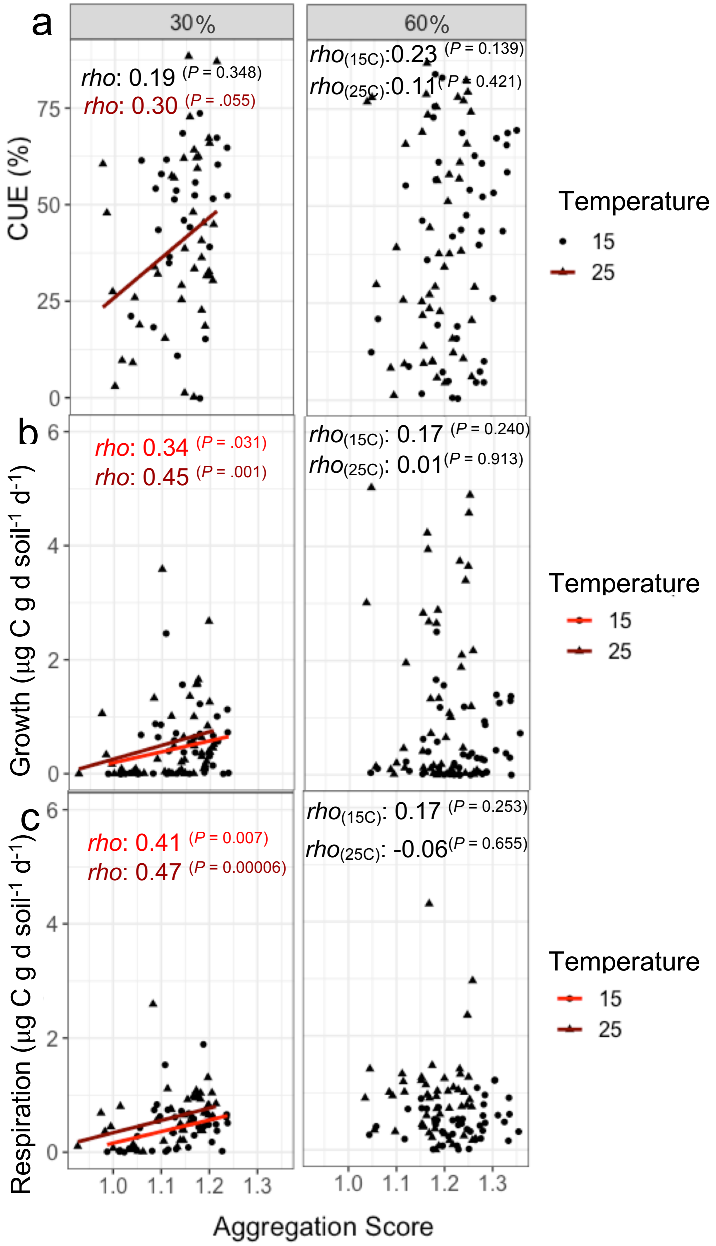

Supplementary Figure 11. Relationship between aggregation score and CUE (a), growth (b) and respiration (c). Significant relationships are evaluated with spearman correlations and shown in light red (15°C) and dark red (25°C). Different moisture treatments shown in different panels: 30% (left) and 60% (right) WHC. The exact *P* value is shown in each panel for each temperature. The number of samples was 40 for 15°C-30%, 49 for 25°C-30%, 45 for 15°C-60 and 50 for 25°C-60%.

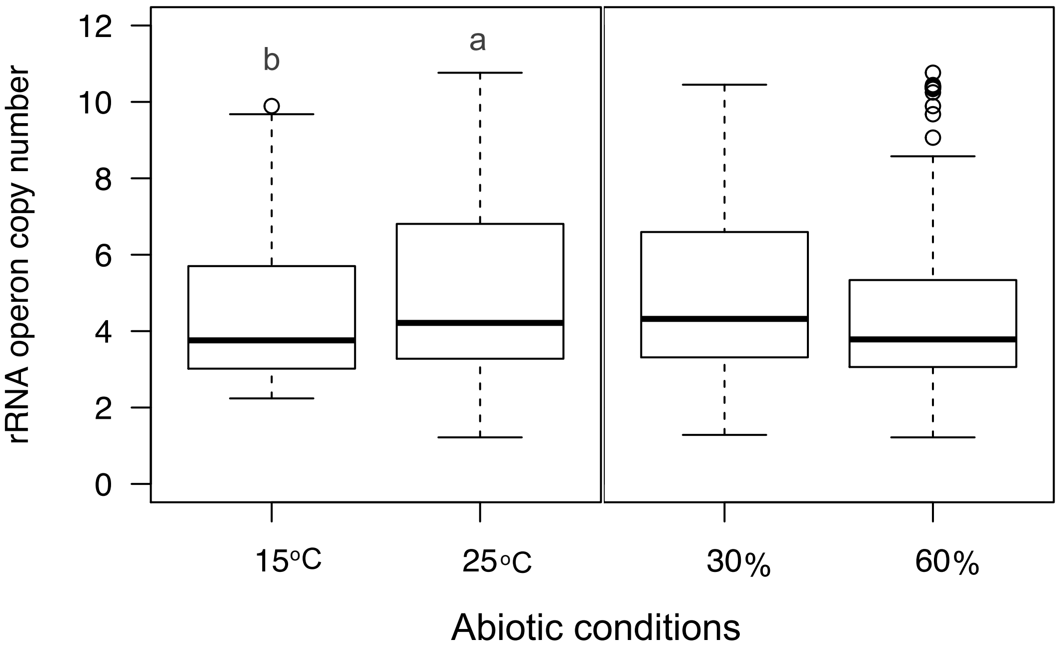

Supplementary Figure 12. Ribosomal RNA operon (rrn) copy number at the different abiotic conditions. Different incubation temperature and moisture are shown in left and right panels, respectively. Significant differences between treatments are indicated by different letters (one-way ANOVA followed by Tukey HSD test, $P < 0.05$, df = 157 , n=176). In the boxplots, whiskers denote the minimum value or 1.5x interquartile range (whichever is more extreme), and box denotes interquartile range. The horizontal line denotes the median. The number of samples was 79 for 15°C, 97 for 25°C, 82 for 30% and 94 for 60% WHC.

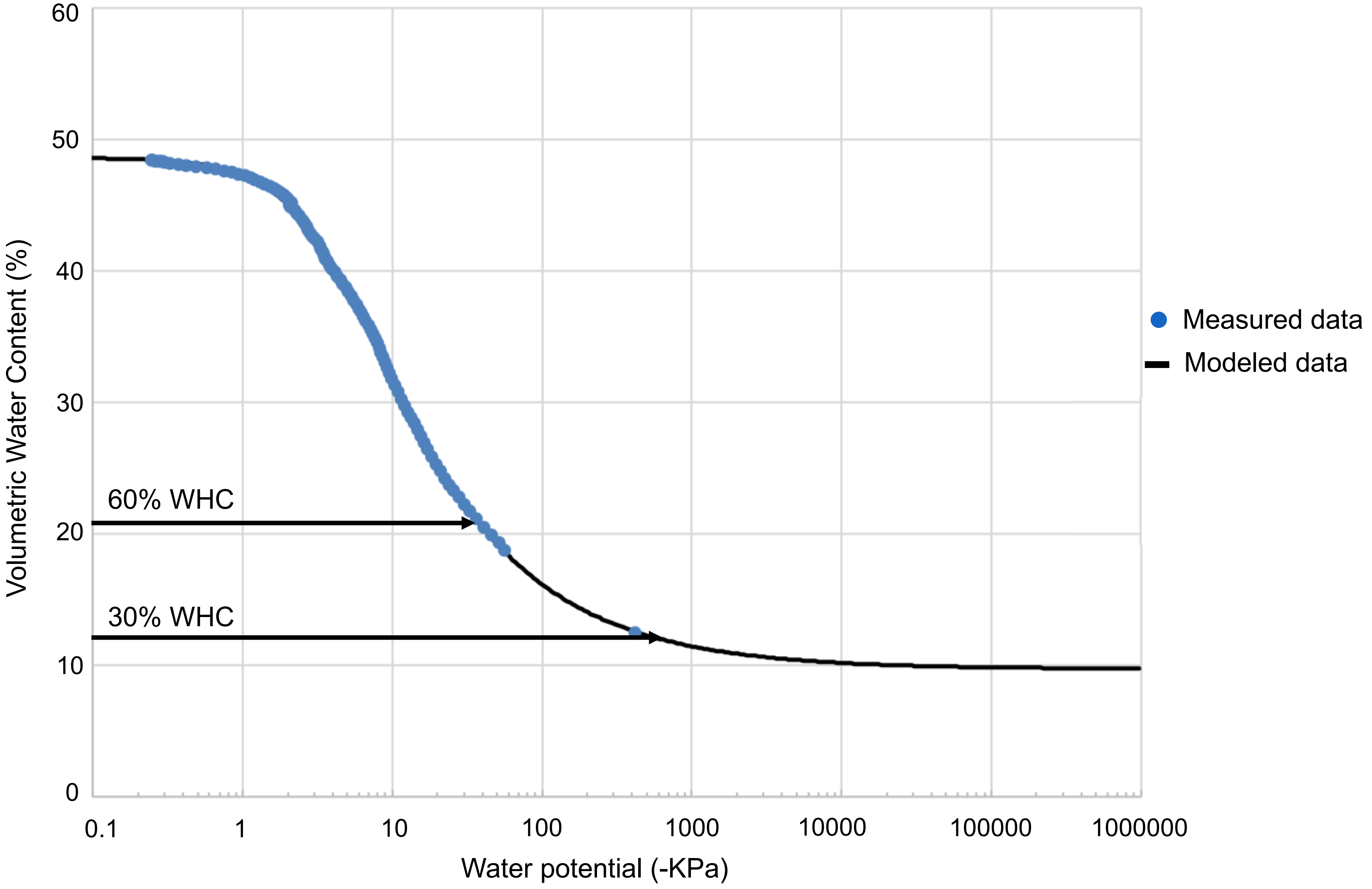

Supplementary Figure 13. Water retention curve in the model soil. Blue points indicate water potential measured by the HYPROP method and the black line is a fitted model to the data based on the van Genuchen model.

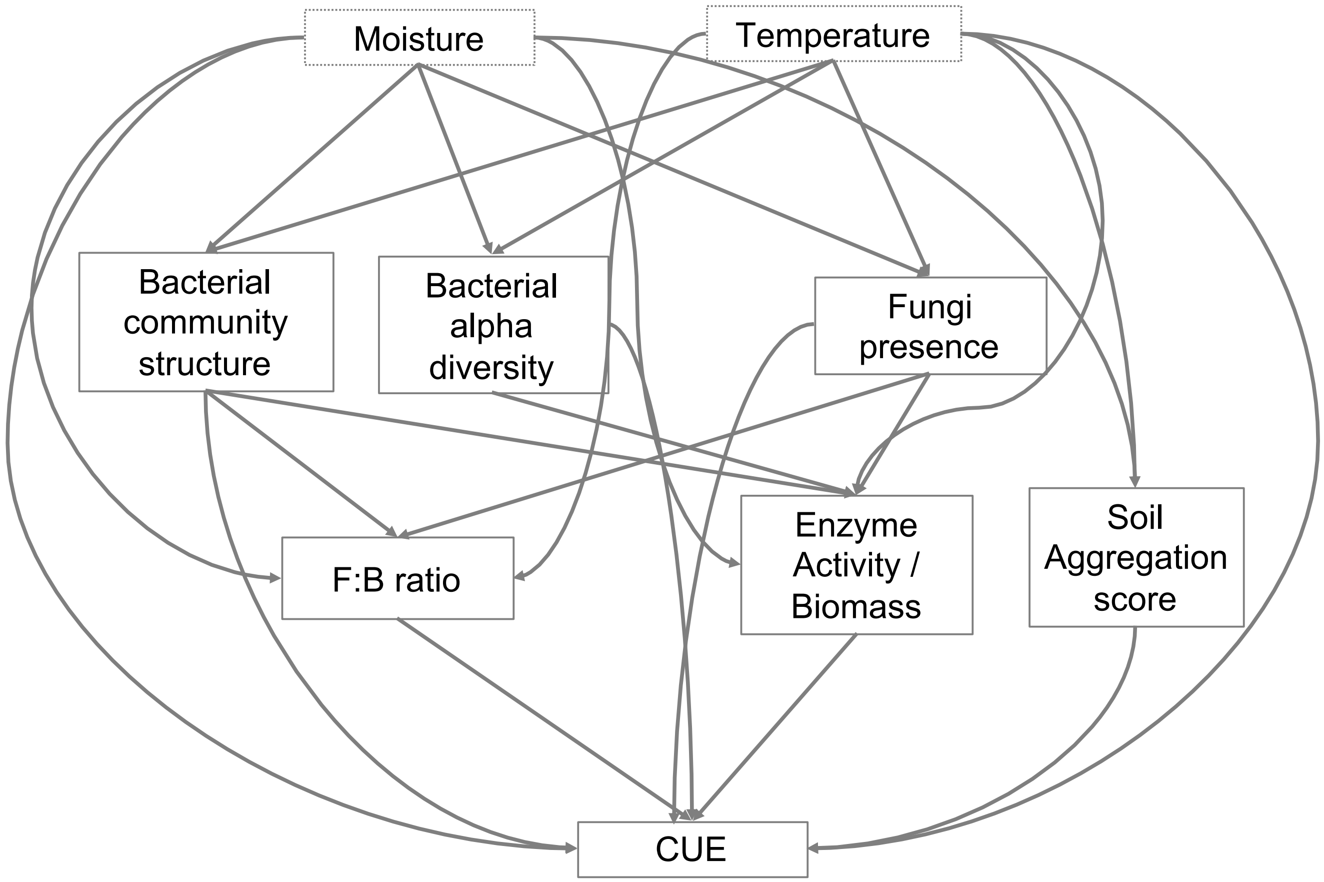

Supplementary Figure 14. Hypothesized path model structure to evaluate abiotic and biotic factors direct and indirect effects of on CUE. We infer that: 1) abiotic factors drive biotic factors and CUE; 2) microbial alpha diversity, community structure and fungi presence are driving the extra-cellular enzymatic activity, the fungi:bacteria ratio and aggregation score; 3) Fungi presence drives the aggregation score and 4) moisture, temperature, microbial alpha diversity and community structure, fungal:bacterial ratio, extra-cellular enzyme activity and aggregation score drives CUE.

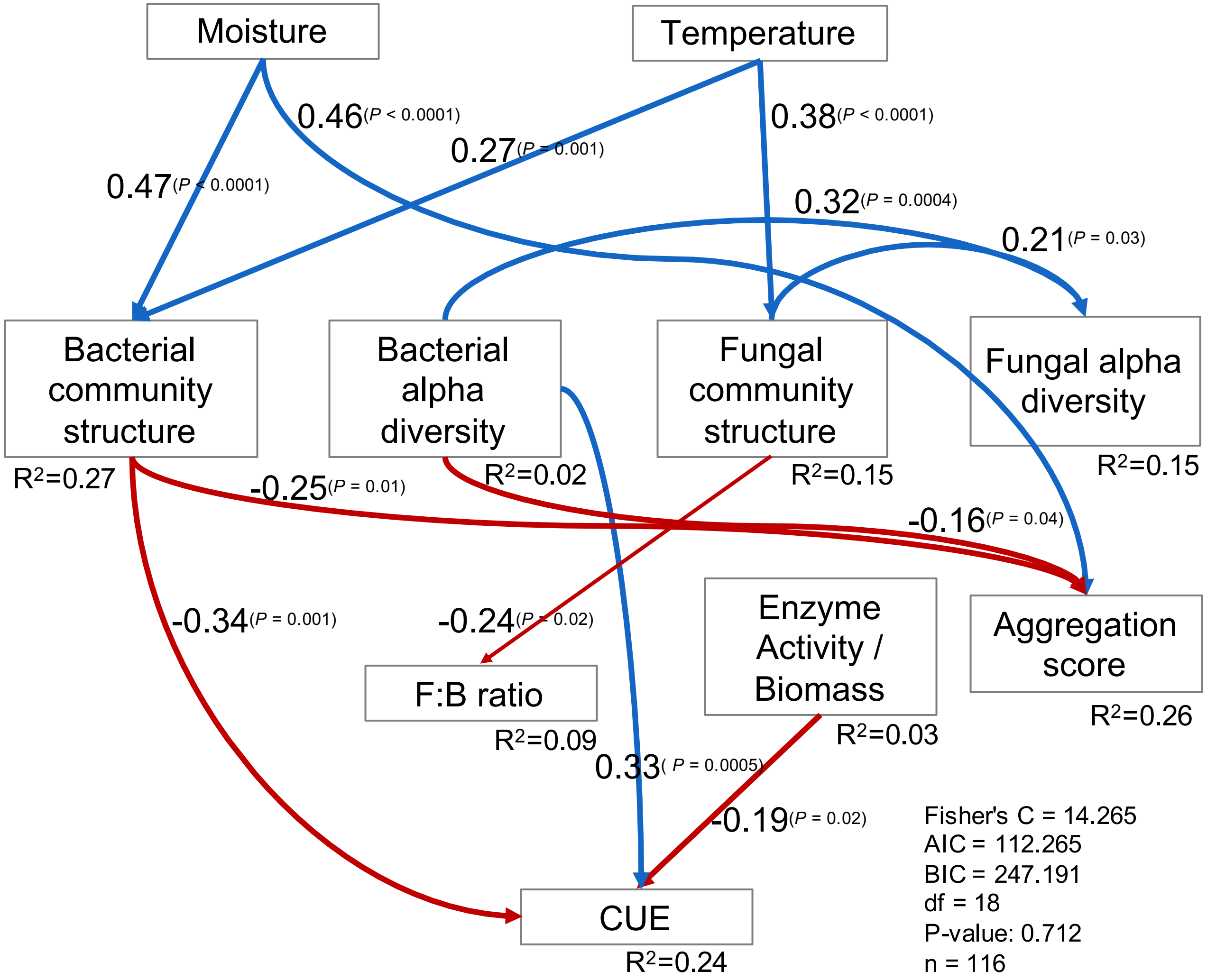

Supplementary Figure 15. Structural equation model showing the relative influence of soil abiotic and biotic factors influence on CUE with fungal community structure and fungal alpha diversity. Significant paths are shown in blue if positive or in red if negative. Exact *P* value is shown for every path coefficient. Amount of variance explained by the model (R^2) are shown for each response variable, and measures of overall model fit are shown in the lower right. Bacterial community structure: axis 1 of NMDS; Bacterial alpha diversity: bacterial phylogenetic diversity index; Fungal community structure: axis 1 of NMDS; fungal alpha diversity: fungal shannon diversity index; F:B ratio: 16S rRNA gene copy number g^{-1} soil: ITS gene copy number g^{-1} soil; Enzyme activity : Biomass: maximum activity recorded for Betaglucosidase/Microbial biomass carbon. CUE: carbon use efficiency; Global goodness-of-fit: Fisher's C.

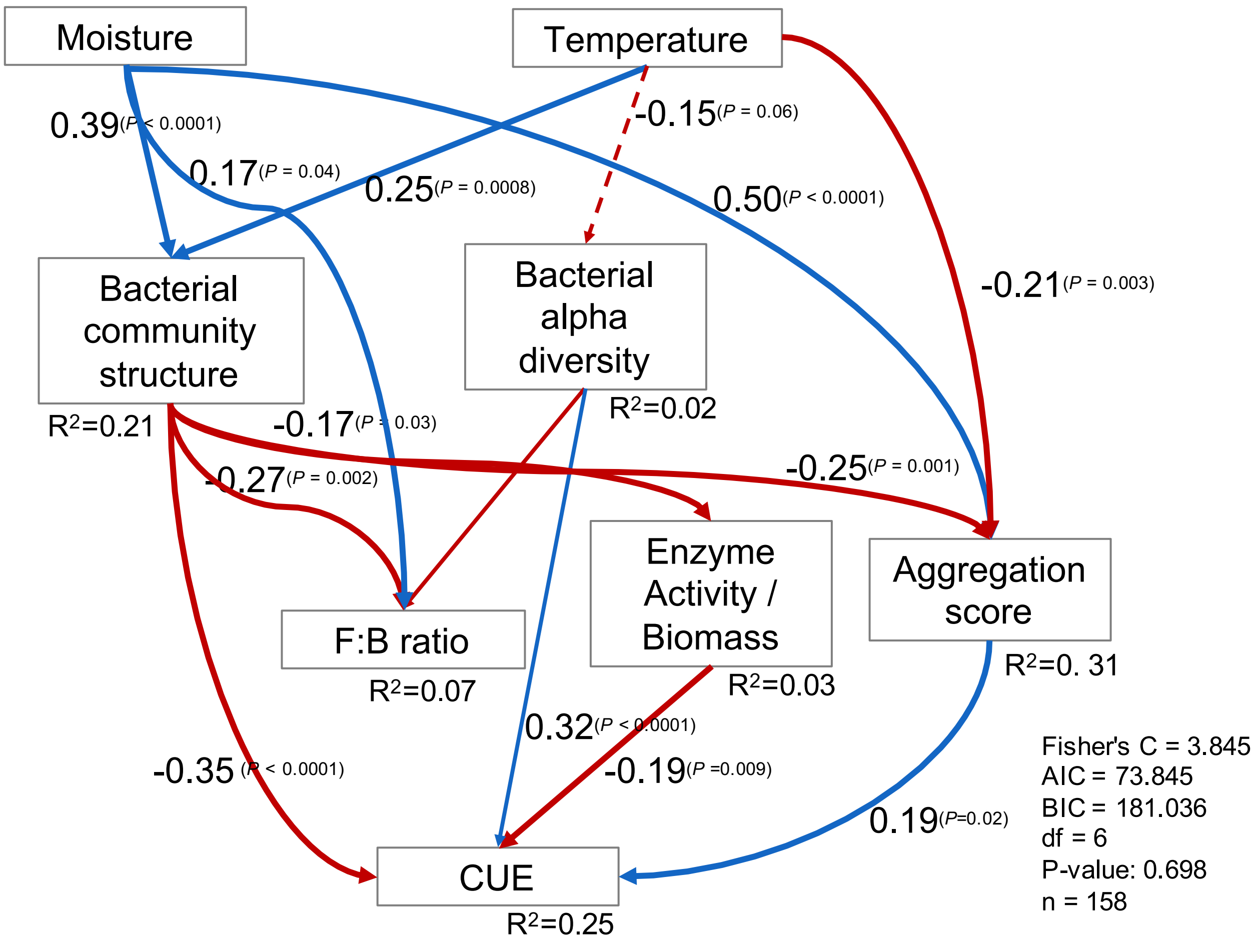

Supplementary Figure 16. Structural equation modeling showing the relative influence of soil abiotic and biotic factors influence on CUE excluding fungal parameters. Significant paths coefficients are shown in blue if positive or in red if negative. Exact *P* value is shown for every path coefficient. Amount of variance explained by the model (R^2) are shown for each response variable, and measures of overall model fit are shown in the lower right. Bacterial community structure: axis 1 of NMDS; Bacterial alpha diversity: bacterial phylogenetic diversity index; F:B ratio: 16S rRNA gene copy number g^{-1} soil: ITS gene copy number g^{-1} soil; Enzyme activity/Biomass: maximum activity recorded for Betaglucosidase/Microbial biomass carbon. CUE: carbon use efficiency; Global goodness-of-fit: Fisher’s C.

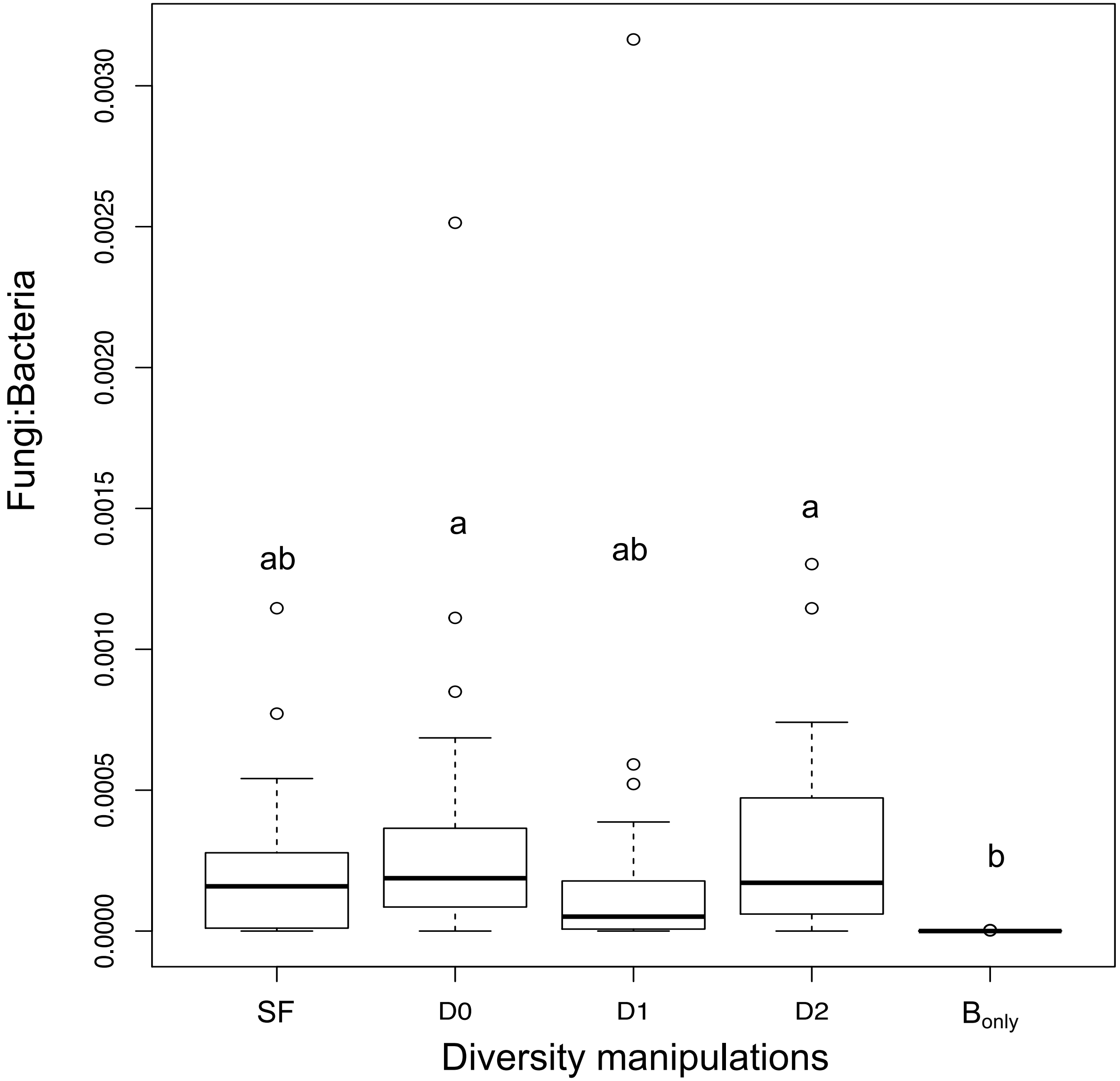

Supplementary Figure 17. Fungi:bacteria ratio of gene copy number by diversity treatment. Significant differences between treatments are indicated by different letters (one-way ANOVA followed by Tukey HSD test, $P < 0.05$, df = 163, n = 168). In the boxplots, whiskers denote the minimum value or 1.5x interquartile range (whichever is more extreme), and box denotes interquartile range. The horizontal line denotes the median. The number of samples was 40 for D0, 39 for D1, 32 for D2, 35 for B_{only} and 22 for SF.

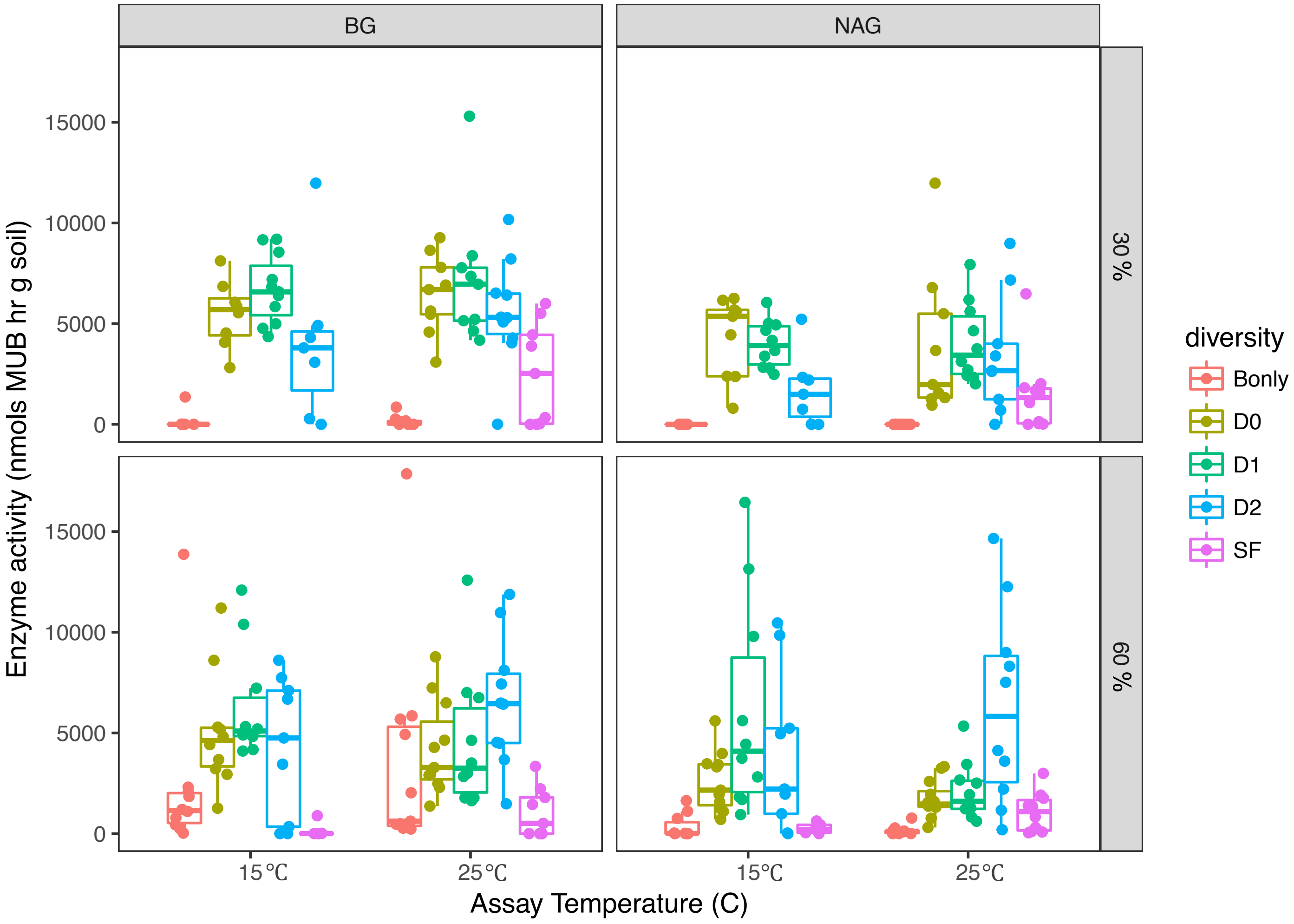

Supplementary Figure 18. Extracellular enzyme activity. Maximum BG and NAG activity for the different diversity treatments, low and high water content treatments, and 15ºC and 25ºC.). In the boxplots, whiskers denote the minimum value or 1.5x interquartile range (whichever is more extreme), and box denotes interquartile range. The horizontal line denotes the median. The number of samples was 40 for D0 and D1, 35 for D2, 38 for Bonly, and 31 for SF.
