# Supplementary material for: Microbial diversity drives carbon use efficiency in a model soil
Source: Nat Commun. 2020 Jul 23;11:3684. doi: 10.1038/s41467-020-17502-z (PMC7378083; doi:10.1038/s41467-020-17502-z)
Supplement: Supplementary file 3 — Reporting Summary [file 41467_2020_17502_MOESM3_ESM.pdf]

nature research

NCOMMS-19-41184A
Corresponding author(s): Domeignoz-Horta, Luiz A. and DeAngelis, Kristen M.

Last updated by author(s): 2020/06/08

# Reporting Summary

Nature Research wishes to improve the reproducibility of the work that we publish. This form provides structure for consistency and transparency in reporting. For further information on Nature Research policies, see Authors & Referees and the Editorial Policy Checklist .

## Statistics

For all statistical analyses, confirm that the following items are present in the figure legend, table legend, main text, or Methods section.

| n/a | Confirmed | |
|---|---|---|
| ☐ | ☒ | The exact sample size (*n*) for each experimental group/condition, given as a discrete number and unit of measurement |
| ☐ | ☒ | A statement on whether measurements were taken from distinct samples or whether the same sample was measured repeatedly |
| ☐ | ☒ | The statistical test(s) used AND whether they are one- or two-sided<br>*Only common tests should be described solely by name; describe more complex techniques in the Methods section.* |
| ☐ | ☒ | A description of all covariates tested |
| ☐ | ☒ | A description of any assumptions or corrections, such as tests of normality and adjustment for multiple comparisons |
| ☐ | ☒ | A full description of the statistical parameters including central tendency (e.g. means) or other basic estimates (e.g. regression coefficient) AND variation (e.g. standard deviation) or associated estimates of uncertainty (e.g. confidence intervals) |
| ☐ | ☒ | For null hypothesis testing, the test statistic (e.g. *F*, *t*, *r*) with confidence intervals, effect sizes, degrees of freedom and *P* value noted<br>*Give P values as exact values whenever suitable.* |
| ☒ | ☐ | For Bayesian analysis, information on the choice of priors and Markov chain Monte Carlo settings |
| ☐ | ☒ | For hierarchical and complex designs, identification of the appropriate level for tests and full reporting of outcomes |
| ☒ | ☐ | Estimates of effect sizes (e.g. Cohen's *d*, Pearson's *r*), indicating how they were calculated |

*Our web collection on statistics for biologists contains articles on many of the points above.*

## Software and code

Policy information about availability of computer code

| | |
|---|---|
| Data collection | No software was used to collect data. |
| Data analysis | The R software (version 3.5.2) was used to perform all analysis. The R code supporting the findings presented here is available from the corresponding authors and from the Open Science Framework Repository (https://osf.io/qmf8z/). |

For manuscripts utilizing custom algorithms or software that are central to the research but not yet described in published literature, software must be made available to editors/reviewers. We strongly encourage code deposition in a community repository (e.g. GitHub). See the Nature Research guidelines for submitting code & software for further information.

## Data

Policy information about availability of data

All manuscripts must include a data availability statement. This statement should provide the following information, where applicable:
- Accession codes, unique identifiers, or web links for publicly available datasets
- A list of figures that have associated raw data
- A description of any restrictions on data availability

The data and R code supporting the findings presented here are available from the corresponding authors on request and from the Open Science Framework Repository (https://osf.io/qmf8z/). The sequencing data is available in the NCBI repository with the identifiers PRJNA556439 and PRJNA556522 for bacteria and fungi, respectively. In the manuscript the data and code avaiability is in L440-444.

# Field-specific reporting

Please select the one below that is the best fit for your research. If you are not sure, read the appropriate sections before making your selection.

# Ecological, evolutionary & environmental sciences study design

All studies must disclose on these points even when the disclosure is negative.

| | |
|---|---|
| Study description | The overall aim of this study was to provide empirical evidence for the response of carbon use efficiency (CUE) to the combined effects of temperature, moisture, diversity loss and distinct community compositions. To do so we used microcosms comprised of model soil inoculated with communities extracted from a temperate deciduous forest soil. The communities were obtained with 5 different inoculum, and incubated under two different temperature (15C or 25C) and two distinct moisture (30 or 60% WHC) treatments with 10 replicates in a full factorial design. Thus: 5 (inoculum) x 2 (temperatures) x 2 (moisture) x 10 (replicates)= 200 samples.<br><br>We also had non-inoculated microcosms which did not receive an inoculum (abiotic controls) with 5 replicates for each temperature (15C or 25C) and moisture (30 or 60% WHC) treatment. Thus, adding up to 20 control samples.<br><br>Moreover, To disentangle the influence of short-term changes in temperature and water content from the effect of distinct microbial communities on CUE, we measured CUE under all different abiotic combinations in a subset of microcosms. For this we choose the more diverse treatment at low moisture (30% WHC) and both temperatures (15C and 25C). Thus: 20 samples x 4 different conditions (15C and 25C and 30 or 60% WHC) = 80 samples. |
| Research sample | Model soil systems provide a platform for controlling specific biotic and abiotic components that play a major role governing soil processes, allowing the isolation of specific components from other confounding variables compared to natural soils. Here, we used a model soil to evaluate how microbial diversity, community structure, moisture and temperature drive carbon use efficiency. Thus, our research samples are model soil microcosms and we previously explained how these microcosms were obtained (section "study description"). |
| Sampling strategy | We had 10 replicates per treatment in a full factorial design. We based this on previous studies (Malik et al., 2018. Land use driven change in soil pH affects microbial carbon cycling processes. Nature Communications; Vogel et al., 2014. Establishment of macro-aggregates and organic matter turnover by microbial communities in long-term incubated artificial soils. Soil Biology Biochemistry; Guenet et al., 2011. A new protocol for an artificial soil to analyse soil microbiological processes. Applied Soil Ecology.). |
| Data collection | At the end of the incubation microcosms were harvested under sterile conditions. Each microcosm was sieved at 2mm and allocated for different assays: 1 g for gravimetric water content, 1.5 g for enzymatic assays, 9 g for microbial biomass carbon (MBC) measurement, 2 g for aggregates formation, and finally 1.2 g was weighed for 18O-H2O-CUE assay and the same DNA extraction was used for sequencing analysis and quantitative real-time PCR (qPCR). The gravimetric water was measured by drying the samples at 65C overnight in an drying oven. The enzymatic assays were performed in a spectramax M2 plate reader coupled with Softmax v6 software. MBC was also quantified colorimetrically using the SpectraMax M2 plate reader. The soil aggregate formation assay was based on mass measurements determined using a laboratory scale. DNA was quantified using PicoGreen DNA stain, and gene copy number was determined on an Eppendorf RealPlex qPCR machine. Respiration was measured using a Quantek instruments IRGA model 906. DNA 18O label incorporation was determined using TC/EA-IRMS (Delta V Advantage, Thermo Fisher, Germany) at the UC Davis Stable Isotope Facility. 16S rRNA and ITS were sequenced in a Ilumina MiSeq machine at the Argonne National Laboratory. Data collection was performed by L.D.H. |
| Timing and spatial scale | The microcosms were incubated for 120 days to allow microbial communities to reach same number of cells per g soil. Because the SF treatment didn't show respiration above abiotic controls until 6 weeks after inoculation, we let this treatment incubate for additional 6 weeks to account for equivalent time of microbial activity. Spatial scale does not apply to this study. Studies that support the timing used in our study: Calderon et al., 2017. Effectiveness of ecological rescue for altered soil microbial communities and functions. ISME; Kallenbach et al., 2016. Direct evidence for microbial-derived soil organic matter formation and its ecophysiological controls. Nature Communications; Guenet et al., 2011. A new protocol for an artificial soil to analyse soil microbiological processes. Applied Soil Ecology.<br><br>Due to the huge number of samples and various assays performed here we randomly sampled 45 microcosms every week to ensure that soil samples would not wait different periods before being analyzed. Thus, in a month we finished to harvest all treatments except the SF treatment which was incubated for additional 6 weeks. The gravimetric water content was determined after sampling; enzymatic assays and microbial biomass carbon were performed the 5 days following sampling; the 18O-H2O-CUE assay was performed 48 hr after sampling and the soil was frozen at the end of incubation to wait for DNA extraction. The aggregate formation assay was performed after two months of sampling. |
| Data exclusions | The SF treatment at 15C and 30% WHC showed no measurable respiration and was thus discarded from this study.<br>Some samples showed negative 18O-atom% excess, which result in negative growth values and therefore were excluded from the analysis. |
| Reproducibility | To reduce stochasticity during microbial communities extraction, all extractions were performed in duplicate and pooled before microcosms inoculation. Moreover we performed technical duplicates for DNA extraction, every microcosms had 2 subsamples receiving 18O-H2O and 2 subsamples receiving 16O-H2O, adding up to 800 DNA extractions. DNA extractions were quantified and subsamples pooled prior to sending the samples to the Stable Isotope Facility. The microbial biomass carbon assay was performed with 3 technical replicates; the aggregate formation assay was performed with 2 technical replicates; qPCR assays were done in duplicate and with two independent assays performed for each gene for each sample and extracellular enzyme activities had 7 replicates for each enzyme type and concentration. The authors did not attempt to replicate the entire experiment. |

| | |
|---|---|
| Randomization | Every week microcosms were randomly allocated within each incubator. Microcosms were also sampled in a random order. |
| Blinding | Data acquisition and analysis was done blindly as every microcosms was assign a specific number and I didn't know to which treatment that specific number corresponded during the data acquisition and analysis. |

Did the study involve field work? ☐ Yes ☒ No

# Reporting for specific materials, systems and methods

We require information from authors about some types of materials, experimental systems and methods used in many studies. Here, indicate whether each material, system or method listed is relevant to your study. If you are not sure if a list item applies to your research, read the appropriate section before selecting a response.

## Materials & experimental systems

| n/a | Involved in the study |
|---|---|
| ☒ | ☐ Antibodies |
| ☒ | ☐ Eukaryotic cell lines |
| ☒ | ☐ Palaeontology |
| ☒ | ☐ Animals and other organisms |
| ☒ | ☐ Human research participants |
| ☒ | ☐ Clinical data |

## Methods

| n/a | Involved in the study |
|---|---|
| ☒ | ☐ ChIP-seq |
| ☒ | ☐ Flow cytometry |
| ☒ | ☐ MRI-based neuroimaging |
